# Surface Energy Balance Based Evapotranspiration Mapping in the Texas High Plains

**DOI:** 10.3390/s8085186

**Published:** 2008-08-28

**Authors:** Prasanna H. Gowda, José L. Chávez, Terry A. Howell, Thomas H. Marek, Leon L. New

**Affiliations:** 1 Conservation and Production Research Laboratory, Agricultural Research Service, U.S. Department of Agriculture, P.O. Drawer 10, Bushland, TX 79012, U.S.A.; E-mails: Jose.Chavez@ars.usda.gov (J.L.C.); Terry.Howell@ars.usda.gov (T.A.H.); 2 Texas AgriLife Research, Texas A&M System, Amarillo, TX 79106, U.S.A.; E-mails: t.marek@tamu.edu (T.H.M.); l-new@tamu.edu (L.L.N.)

**Keywords:** Ogallala Aquifer Region, irrigation scheduling, semi-arid environment

## Abstract

Agriculture on the Texas High Plains (THP) uses approximately 89% of groundwater withdrawals from the Ogallala Aquifer. Consequently, groundwater levels are declining faster than the recharge rate. Therefore, efficient agricultural water use is essential for economic viability and sustainability of the THP. Accurate regional evapotranspiration (ET) maps would provide valuable information on actual crop water use. In this study, METRIC (Mapping Evapotranspiration at High Resolution using Internalized Calibration), a remote sensing based ET algorithm, was evaluated for mapping ET in the THP. Two Landsat 5 Thematic Mapper images acquired on 27 June (DOY 178) and 29 July (DOY 210) 2005 were used for this purpose. The performance of the ET model was evaluated by comparing the predicted daily ET with values derived from soil moisture budget at four commercial agricultural fields. Daily ET estimates resulted with a prediction error of 12.7±8.1% (mean bias error ± root mean square error) on DOY 178 and -4.7±9.4% on DOY 210 when compared with ET derived from measured soil moisture through the soil water balance. These results are good considering the prevailing advective conditions in the THP. METRIC have the potential to be used for mapping regional ET in the THP region. However, more evaluation is needed under different agroclimatological conditions.

## 1. Introduction

The Ogallala Aquifer, one of the largest freshwater aquifers in the world, has been the main source of water supply for the Texas High Plains (THP) population in the U.S. It is being depleted at an unsustainable rate [1]. Irrigation alone uses approximately 89% of the water pumped from the Ogallala Aquifer in the THP. A change in water storage in the Ogallala Aquifer beneath the Texas High Plains from predevelopment to 2003 was about -164.1 km^3^ (-5.2 km^3^ from 2002 to 2003) with an average area-weighted water-level change of -10.6 m (-0.37 m from 2002 to 2003) and more than 3.8 million ha of land with water level declines of 7.6 m or more [2, 3]. For this reason and considering the positive trends in population growth in the THP, there is a tremendous emphasis for achieving greater efficiency in irrigation water management for agriculture.

Improvement in irrigation water management is achieved when the beneficial crop water use is accurately quantified in time and space for decision making in terms of timing and amounts of water to apply. Remote sensing (RS) based evapotranspiration (ET) methods are found to be useful for estimating crop water use in both time and space.

Numerous RS algorithms such as METRIC (Mapping Evapotranspiration at high resolution with Internalized Calibration) [4,5], SEBAL (Surface Energy Balance for Land) [6,7], and a two-source energy balance model (TSM) [8], among others, have been developed in an effort to spatially estimate crop water consumption or ET and are being tested around the world. Most of these algorithms mainly solve the energy balance of the land surface for latent heat flux (LE) at the time of satellite or airborne RS system overpass. Extrapolation of instantaneous LE to daily ET and interpolation of daily ET estimations between satellite overpasses to get seasonal values is usually done using locally available weather data. A presentation of numerous RS algorithms for ET estimation along with a discussion on their present status and challenges can be found in the literature [9, 10]. For instance, they indicated that the TSM model yielded instantaneous surface heat fluxes with errors within 10-12%, although this model demands several crop and micro-meteorological data that in many circumstances are very difficult to obtain. On SEBAL algorithm, they explained that a typical daily ET prediction accuracy was 85% or that errors ranged from 2.7 to 35% with an overall average bias of 18.2% under a variety of climatic/environmental conditions. Meanwhile, METRIC appeared to have an advantage over SEBAL under advective conditions because it uses hourly (or shorter period) alfalfa reference ET (ET_r_) instead of the “instantaneous” (30-min or hourly) available energy [net radiation (R_n_) minus soil heat flux (G)], estimated for the time of remote sensing system overpass, in the calculation of the evaporative fraction [EF = LE/(R_n_-G)]. In addition, METRIC uses the instantaneous to daily ET extrapolation method called alfalfa reference ET fraction (ET_r_F = LE/ ET_r_), which employs wind speed and air temperature that according to [4, 5] better incorporate local/regional surface/environmental conditions than the evaporative fraction of other remote sensing ET algorithms.

METRIC's ET estimation errors were reported to be anywhere from 10 to 20% for daily estimates and as low as 4 to 1% for seasonal ET estimates [9], requiring solar radiation, air temperature, vapor pressure and wind speed measurements from weather stations (WS). Therefore, the attributes presented by METRIC make it very attractive for mapping ET under the advective conditions.

The THP is a semi-arid region with heterogeneous landscapes in which irrigated fields are usually surrounded by dryland crops, fallow or rangeland. Therefore, the advection of sensible heat flux from dry surfaces, both local and regional, is a significant source of energy that has a major impact on ET from irrigated cropland by augmenting ET in excess of the available energy (net radiation minus soil heat flux) rather frequently. For example, an average ET rate of 11.3 mm d^-1^ for an irrigated alfalfa in Bushland, Texas was reported, with ET for some days exceeding 15 mm d^-1^ due to regional advection [11].

The main objective of this study was to assess the ability and usefulness of METRIC for mapping regional ET in the THP. We selected METRIC as an ET mapping tool to be applied and evaluated in the THP since it could be an algorithm that works better under advective conditions and requires minimal ground data.

## 2. Materials and Methods

### 2.1. Study Area

**Figure 1.**
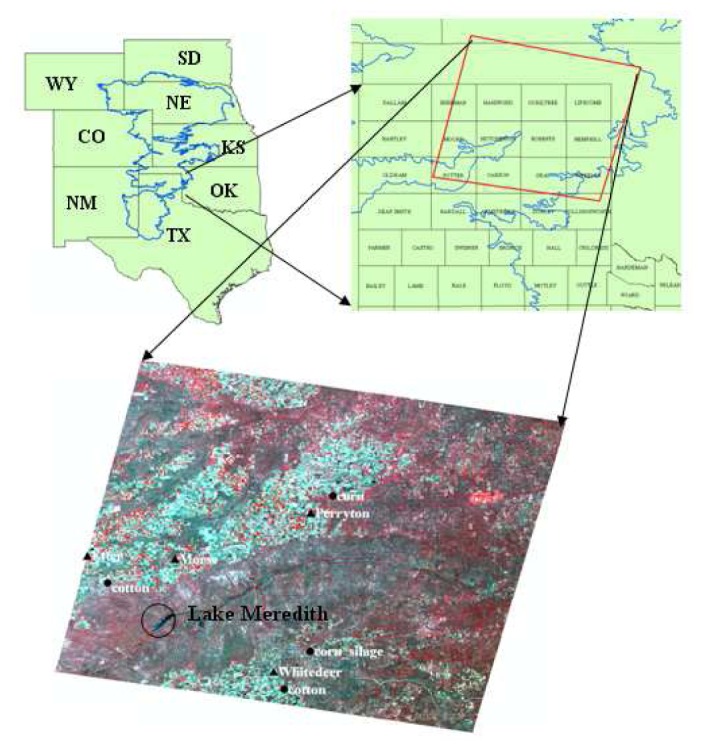
Ogallala Aquifer Region coverage (blue boundary), Landsat 5 coverage area (red rectangle) and false color Landsat 5 image showing location of grass reference weather stations (solid triangles), crop fields containing soil moisture probes (solid circles) and Lake Meredith (empty circle), in the Texas High Plains.

This study was focused on the portion of the THP Region in the south-central U.S. covered by Landsat 5 Thematic Mapper (TM) scene with a path/row of 30/35. The TM scene comprised of 11 counties in the THP (Figure 1), underlain by the diminishing Ogallala Aquifer. The soils are mainly Pullman clay loam and Sherm silty clay loam [12], with nearly level to gently sloping fields occupying nearly all of the cropland. Wind direction is predominantly from the southwest direction. The land use/cover in the study area consists of crops, mesquite shrubs (grassland), mesquite brush, sandsage (Harvard Shin oak brush), buffalo grass (grassland), cottonwood-hackberry-salt cedar brush/wood, and mesquite-juniper brushes [13].

More detailed analysis was concentrated on Ochiltree County, located closer to the center of the scene, where ground truth data on crop types were acquired as part of another remote sensing study. The Ochiltree County area is about 234,911 ha, with more than 45% of the land under row crop production. Annual average precipitation is about 562 mm, while about 890 mm of water are needed to grow grain corn (330 mm of seasonal rainfall), 670 mm for cotton (280 mm), 690 mm for grain sorghum (248 mm), 762 mm for soybean (254 mm), and 457 mm for winter wheat (142 mm) [14]. Values in parenthesis show rainfall amounts during the corresponding cropping season. About 66% of the cropland in Ochiltree County is irrigated with groundwater from the Ogallala Aquifer. Sorghum, wheat and corn are the major crops in the county. Other crops include soybean, cotton, sunflower, and oats.

### 2.1. METRIC

In METRIC, ET is computed as a residual from the land surface energy balance equation as an instantaneous ET or latent heat flux (LE) at the time of the satellite overpass, as shown in Equation (1).

(1) $$\text{LE}={\text{R}}_{\text{n}}-\text{G}-\text{H}$$

where R_n_ is net radiation (W m^-2^), G is the soil heat flux (W m^-2^) and H is the sensible heat flux (W m^-2^). LE is converted to ET (mm h^-1^ or mm d^-1^) by dividing it by the latent heat of vaporization (λ_LE_) [λ_LE_ = 2.501 − 0.00236 (T_a_), MJ kg^-1^ for T_a_ in °C], density of water (ρ_w_; ∼1.0 Mg m^-3^), and an appropriate time constant. The sign convention for the different flux terms in Equation (1) is positive away from the surface (towards the atmosphere) for LE and H and positive towards the surface for R_n_ and G. R_n_ is calculated using surface reflectance and surface radiometric temperature (T_s_) derived from the satellite imagery and near surface vapor pressure from a nearby WS. R_n_ is the result of the surface energy budget between short and long wave radiation terms described as:

(2) $${\text{R}}_{\text{n}}={\text{R}}_{\text{s}}↓-\alpha {\text{R}}_{\text{s}}↓+{\text{R}}_{\text{L}}↓-{\text{R}}_{\text{L}}↑-(1-\varepsilon_{\text{o}}){\text{R}}_{\text{L}}↓$$

where R_s_↓ is incoming shortwave radiation (W m^-2^), which in METRIC is estimated using extraterrestrial radiation. In this study, R_s_↓ was measured with a pyranometer. α is surface albedo (dimensionless), R_L_↓ is incoming long wave radiation (W m^-2^) or downward thermal radiation flux originated from the atmosphere which can be estimated using the Stefan-Boltzmann equation and near surface air temperature as well as vapor pressure for air emissivity. In METRIC, clear-sky R_L_↓ is estimated using broad band atmospheric transmissivity for short wave radiation (used to estimate air emissivity), and T_s_ in place of air temperature. R_L_↑ is outgoing long wave radiation (W m^-2^), it is calculated using the Stefan-Boltzmann constant (5.67 × 10^-8^ W m^-2^ K^-4^), the remotely sensed T_s_ and ε_o_, which is broad-band surface thermal emissivity (dimensionless). This last term is based on soil and vegetative broad band thermal spectral emissivities [function of LAI (Leaf Area Index) or NDVI (Normalized Difference Vegetation Index)], and it can be calculated using empirical equations [15]. The (1- ε_o_)R_L_↓ term represents the fraction of incoming long wave radiation reflected from the surface. Surface albedo is the term that is a function of reflectance values in the shortwave portion of the electro-magnetic spectrum.

Soil heat flux (G) was modeled as a function of R_n_, vegetation index, surface temperature, and surface albedo for near midday values [16]:

(3) $$\text{G}=(({\text{T}}_{\text{s}}-273.15)(0.0038+0.0074\;\alpha )(1-0.98\;{\text{NDVI}}^{4})){\text{R}}_{\text{n}}$$

Sensible heat flux (H) is defined by the bulk aerodynamic resistance equation, which uses aerodynamic temperature and aerodynamic resistance to heat transfer:

(4) $$\text{H}=\rho_{\text{a}}\;{\text{Cp}}_{\text{a}}\;({\text{T}}_{\text{aero}}-{\text{T}}_{\text{a}})/{\text{r}}_{\text{ah}}$$

where ρ_a_ is air density (kg m^-3^), Cp_a_ is specific heat of dry air (1004 J kg^-1^ K^-1^), T_a_ is average air temperature, (K), T_aero_ is average aerodynamic temperature (K), which is defined for a uniform surface as the air temperature at the height of the zero plane displacement (d, m) plus the roughness length (Z_oh_, m) for sensible heat transfer, and r_ah_ is aerodynamic resistance (s m^-1^) to heat transfer from Z_oh_ to Z_m_ [height of wind speed (U, m) measurement].

In METRIC, H is estimated without needing to know the air temperature or the aerodynamic temperature value; instead a temperature difference (dT), function of T_s_, is used as:

(5) $$\text{H}=\rho_{\text{a}}\;{\text{Cp}}_{\text{a}}\;\frac{\text{dT}}{{\text{r}}_{\text{ah}}}$$

where r_ah_ is calculated between two near surface heights, z_1_ and z_2_ (generally 0.1 and 2 m) using U extrapolated to some blending height above the ground surface (typically 100 to 200 m) and an iterative stability correction scheme for atmospheric heat transfer based on the Monin-Obhukov stability length scale (L__MO_, similarity theory [17]). In this study, a height of 200 m was used in the calculation of distributed friction velocity (u*), a term utilized in the estimation of r_ah_. This height was chosen considering the recorded high horizontal U at 2 m height was 7.0 m s^-1^ (average value for the four WS) on DOY 178 and 3.05 m s^-1^ on DOY 210. It would require a higher elevation for the logarithmic wind profile to produce a near constant U. In addition, u* computation utilizes the roughness length for momentum transfer (Z_om_). In METRIC, Z_om_ is estimated as 0.018 times LAI and by limiting its minimum value on bare soils to 0.005 m. According to [4], this model is apparently suitable for crop heights up to 1.0 m. Since irrigated corn and silage sorghum were taller than 1.0 m at the time of image acquisition in the NTHP area, we modified the Z_om_ model to “0.005 + 0.02 LAI.” This local calibration of Z_om_ was performed using ground readings of crop height (h_c_, m) and the relationship between Z_om_ and h_c_ found in the literature [18].

As shown in [4, 5], dT (K) represents the near surface temperature difference between z_1_ and z_2_, and that the indexing of dT to T_s_ does not rely on absolute values of T_s_, which reduces the error substantially in calculating H; “insofar as dT is actually linear in T_s_.” Equation (6) characterizes the relationship of dT to T_s_ as defined in [6].

(6) $$\text{dT}=a+b\;{\text{T}}_{\text{s}}$$

where *a* and *b* are empirically determined constants.

The determination of *a* and *b* in Equation (6) involves locating a hot (dry) pixel (in a fallow agricultural field) with high T_s_ value and a cold (wet) pixel with a low T_s_ value (typically one in an irrigated agricultural setting) in the remote sensing image. Once these pixels have been identified, the energy balance of Equation (1) can be solved for H_cold_ and H_hot_ as:

(7) $$H_{\text{cold}}={\left(R_{n}-G\right)}_{\text{cold}}-{\text{LE}}_{\text{cold}}$$

(8) $$H_{\text{hot}}={\left(R_{n}-G\right)}_{\text{hot}}-{\text{LE}}_{\text{hot}}$$

where H_hot_ and H_cold_ are the sensible heat fluxes for the hot and cold pixels respectively. The hot pixel is defined as having no latent heat flux (i.e., all available energy is partitioned to H), although LE_hot_ may be calculated according to a soil water budget if significant rainfall has occurred in the last couple of weeks before the image acquisition date. The cold pixel is assumed to have latent heat flux equal to 1.05 times [5] that expected for a tall reference crop (i.e., alfalfa), thus LE_cold_ = 1.05 ET_r_ λ_LE_, where ET_r_ is the hourly (or shorter time interval) tall reference (like alfalfa) ET calculated using the standardized ASCE Penman-Monteith equation [19]. A coefficient of 1.05 was used to estimate LE_cold_ as the cold pixel typically have an ET rate of 5% larger than that for the reference ET (ET_r_) due to wet soil surface beneath a full vegetation canopy that will tend to increase the total ET rate [4, 5].

The hot pixel was chosen after careful screening of fallow/bare agricultural fields displaying high temperatures, high albedo and low biomass (LAI). The cold pixel was determined on the basis of low temperature (approximated by the Lake Meredith water surface temperature; Figure 1), high biomass, and albedo of approximately 0.18-0.24.

With the calculation of H_hot_ and H_cold_, Equation (5) was inverted to compute dT_hot_ and dT_cold_. The *a* and *b* coefficients were then determined by fitting a line through the two pairs of values for dT and T_s_ from the hot and cold pixels. The values of *a* and *b* were initial values that were used in an iterative stability correction scheme which after some iterations showed numerical convergence and the *a* and *b* coefficient for each iteration were then used to obtain the final stability corrected H image.

The instantaneous LE image was obtained using Equation (1) and it was converted to ET_i_ map in mm h^-1^ by dividing it by *λ*_LE_ and ρ_w_. In METRIC, *λ*_LE_ is calculated substituting T_a_ by T_s_.

(9) $${\text{ET}}_{\text{i}}=3600\;\text{LE}/(2.501-0.00236\;({\text{T}}_{\text{s}}-273.15))\;10^{6}$$

Reference ET fraction (ET_r_F) is the ratio of ET_i_ to the reference ET_r_ that is computed from WS data for overpass time. The WS information is explained in a subsequent section. Finally, the computation of actual daily or 24-h ET (ET_d_), for each pixel, is performed as:

(10) $${\text{ET}}_{\text{d}}={\text{ET}}_{\text{r}}\text{F}\times {\text{ET}}_{\text{r}}24$$

where ET_r_24 is the cumulative 24-h ET_r_ for the day (mm d^-1^), based on WS data.

### 2.2. Data

Two Landsat 5 TM satellite images were obtained in 2005 for estimating ET, one on DOY 178 (June 27) and another on DOY 210 (July 29). The overpass time for both images was 17:07 GMT (11:07 AM CST in the U.S.). The satellite path/row was 30/35, where the image scene center coordinates were Latitude 36.048° N and Longitude 100.910° W. The image pixel size was 30 m for TM bands 1, 2, 3, 4, 5 and 7 and 120 m for TM band 6 (thermal band). However, the image supplier had resampled TM band 6 to a 30 m pixel size. Figure 1 shows the satellite image in false color (TM bands 4, 3, and 2).

For the calculation of the alfalfa based ET_r_ and ET_r_24, four reference WS identified within the geographic coverage of the satellite scene, were used. These stations were: Perryton, Etter, White Deer, and Morse (Figure 1). The WS are part of both the Texas High Plains ET Network (TXHPET) and weather data can be accessed from their websites [20, 21]. The TXHPET and TNPET reported hourly and daily weather data as well as the grass (ET_o_) and alfalfa (ET_r_) reference ET calculated using the standardized ASCE Penman-Monteith method. The WS grass cover types were: native pasture (Perryton), Buffalo grass (Etter), native pasture (White Deer), and native grass (Morse), respectively.

Soil water content measurements were used to derive ET for comparison with RS estimates. These measurements were taken as part of another study in four different, commercially operated, agricultural fields (Figure 1). These fields included a fully irrigated grain corn field, an irrigated silage corn field, an irrigated cotton field, and a cotton field under limited irrigation. Soil water was monitored by the Texas Cooperative Extension Service (Texas A&M University System) by means of a KS-D1 Gypsum block meter (Delmhorst Instruments Company, Towaco, N.Y.) connected to GB-1 Gypsum blocks sensors. The blocks were installed at a depth of 0.3, 0.6, and 0.9 m, respectively, and readings were recorded at least twice each week [14]. Although Gypsum block sensors are considered somewhat unreliable [22], they perform well in fine texture soils [23, 24]. In our case, most soils were clay loam and thus it is expected the sensors to perform better than reported in the literature. The date, number and amount of individual irrigations were recorded and calculated using deep well water flow deliveries [14]. Rainfall data measured at the site were used. An explanation on the derivation of volumetric soil water content and soil water depths from the meter readings can be found in Appendix A.

### 2.3. Radiometric and Atmospheric Calibration of Satellite Images

The spatial mapping of ET for the NTHP was done using the METRIC algorithm. For this purpose, the satellite image was calibrated, the digital number (DN) was first converted into radiance (L_b_), for each band by means of calibration coefficients (L_b_= (gain × DN) + bias) provided by the image supplier. Then, to obtain “at-satellite” or at “Top-of-the-Atmosphere” (TOA) reflectance values, for the short-wave bands, the detected radiance at the satellite (for each band) was divided by the incoming energy (radiance) in the same short-wave band. This incoming radiance is a function of mean solar exo-atmospheric irradiance, solar incidence angle, and the inverse square of the relative earth-to-sun distance. Detailed steps on the Landsat 5 TM radiometric calibration procedure can be found in [25]. Subsequently, the at-surface reflectance values were computed after applying atmospheric interference corrections, on the TOA reflectance image, using calibrated atmospheric transmittance and path reflectance functions found in [26]. These functions correct TOA reflectance images for scattering and absorption of incoming and reflected solar radiation from the surface based on a simplified atmospheric correction function that requires only point measurements or estimates of near surface vapor pressure (e_a_) [26]. Similarly, T_s_ was obtained after converting DNs in TM band 6 image to radiance and then to corrected thermal radiance following [4, 5] and finally to T_s_ following [25]. Basically, corrections applied to the thermal image included the use of narrow band transmissivity to obtain corrected radiance values and then narrow band emissivity to obtain T_s_ with a general atmospheric correction for clear sky atmospheric conditions.

### 2.4. METRIC ET Verification

METRIC ET estimates (ET__estimated_) were compared with ET derived from soil water content readings (ET__observed_) at four different locations by means of the soil water balance (SWB):

(11) $${\text{ET}}_{\_\text{observed}}=\theta_{\text{i}-1}-\theta_{\text{i}}+I+\text{P}$$

where θ_i_ is the soil water (depth equivalent, mm) in the root zone at the beginning of day “i”, θ_i-1_ is the soil water equivalent (mm) in the root zone at the beginning of day “i-1”, i.e. the previous day, *I* is net irrigation depth (mm), and P is the rainfall (mm). In this study, *I* was estimated from measured volumetric water deliveries, center pivot area and by assuming irrigation water application efficiency (IE_a_) of 90%; the IE_a_ value was published for LESA (Low Elevation Spray Application) Center Pivot irrigation systems as common for the NTHP area [27]. The SWB calculations were performed over a period of 3 or 4 days depending on the number of readings per week. θ_i_ computation from the soil water content data at three depths is explained in Appendix B.

Results of the comparison of ET using METRIC and ET from soil water content measurements, for each field, were reported as absolute differences and in percent errors according to:

(12) $$\text{Percent Error}(\%)=({\text{ET}}_{\text{estimated}}-{\text{ET}}_{\text{observed}})\times 100/{\text{ET}}_{\text{observed}}$$

A more comprehensive evaluation of ET estimation errors (comparison of estimated/measured ET_d_) was carried using the Mean Bias Error (MBE) and the Root Mean Square Error (RMSE). These are the mean and standard deviation errors respectively.

## 3. Results and Discussion

### 3.1. Surface Temperature

Derived surface temperatures within the THP area, covered by the Landsat 5 TM scene, ranged from 18.6 to 34.9 °C, a difference of 16.3 °C on DOY 178; 18.4 °C to 42.0 °C, a difference of 23.6 °C on DOY 210. This spatial variation in surface temperature highlights the uniqueness of the cropping conditions in the THP where irrigated/non-irrigated crop fields intermix with fallow/bare soil lands and where local and regional advection may increase ET rates by augmenting the advected sensible heat flux. In a separate study in Bushland, TX (Figure 1), it was found that an average of 61% of the total ET could be attributed to advective sensible heat for an average U of 4.4 m s^-1^ [11]. In this study, U at the time of satellite overpass was 7.0 m s^-1^ on DOY 178 and 3.05 m s^-1^ on DOY 210 (Perryton WS). In addition, more than half of the area was not irrigated and some irrigated cotton, soybean and sorghum fields were at the very early growth stage (LAI<1.5) with partial canopy cover, a situation that may have contributed to local advective conditions.

### 3.2. Net Radiation

Average R_n_ for the entire satellite scene was 616.0 and 642.6 W m^-2^ for DOY 178 and 210, respectively; with non-water stress high biomass (LAI > 3.0) fields depicting higher R_n_ values. Bare soils showed lower R_n_ values, 500-550 W m^-2^ and 530-590 W m^-2^ on DOY 178 and 210, respectively.

### 3.3. Soil Heat Flux

On DOY 178, the average G value for the entire satellite scene was 87 W m^-2^. Soil heat flux ranged from 80 to 100 W m^-2^ for bare soils and 25 to 40 W m^-2^ for high biomass crops. However, average G value for the satellite scene acquired on DOY 210 was 116.4 W m^-2^, i.e. 34% higher than that G on the DOY 178 image; with a range of 120-140 W m^-2^ for bare soils and 22-50 W m^-2^ for high biomass crops. Increase in the average G value in the later image was probably due to drier surface conditions.

### 3.4. Sensible Heat Flux

In the determination of H, the colder (wet) pixel was located in an irrigated corn field having a surface temperature of 18.6°C (291.7 K) on both analyzed images. This temperature was about half a degree higher than the water temperature in Lake Meredith (Figure 1) indicating that the corn field was using all the available energy (AE = R_n_-G) in the ET process. The hotter (dry) pixel was found in a nearby fallow dry field. Table 1 reports ET_r_F, T_s_, R_n_, G, Z_om_, and U at a blending height of 200 m for wet and dry pixels. For the hot pixel, ET_r_F was assumed zero (0), i.e. no ET on DOY 178 and 210, since there was no significant rainfall events occurred within the two weeks prior to the satellite overpasses.

Values in the upper portion of Table 1 were used in the initial estimation of dT and H, for both hot and cold pixels under neutral atmospheric conditions. Initial H and dT values were subsequently adjusted for the unstable atmospheric conditions, encountered for DOY 178 and 210, using the Monin-Obukhov length scale iterative method. Table 1 reports the resulting final values for r_ah_, horizontal friction velocity (u*), L__MO_, dT, LE, and H for both cold and hot pixels.

Setting ET_r_F to 1.05 for the cold pixel resulted in a negative H value, meaning that the air temperate was higher than the corn canopy temperature, thus extra heat was brought in by local and regional advection. The advection scenario was discussed early in this results section. This extra heat produced an H (cold pixel) that enhanced LE beyond available energy (633.9 W m^-2^) by 24.4% on DOY 178 and by 2.5% on DOY 210. These results are in agreement with results reported in another study [11].

### 3.5. Daily ET

Average ET_d_ was 5.7 mm d^-1^ with a mode and maximum values of 6.9 and 14.5 mm d^-1^, respectively, for the entire satellite scene on DOY 178. Using all four WS data, the average ET_r_24 was 13.5 mm d^-1^ and ET_r_ was 1.1 mm h^-1^ at the time of satellite overpass. On DOY 210, the ET_r_24 and ET_r_ were 9.7 mm d^-1^ and 0.95 mm h^-1^, respectively. These ET_r_ and ET_r_24 values were used in the internalized calibration (scaling) of ET.

Estimated ET_d_ for a fully irrigated corn, on DOY 178, compared reasonably well with ET_observed_ (Table 2). There was an overestimation of 2.0 mm d^-1^ or an error of 17.1%. The estimation error was 17.7% for the irrigated silage corn field, 3.4% for irrigated cotton, and –71.4% for the limited irrigated cotton, which had a very small ET rate of just 1.4 mm d^-1^. Overall, for DOY 178 the average ET estimation errors were (MBE±RMSE) 1.1±0.9 mm d^-1^ or 12.7±8.1% excluding the ET data from the limited irrigated cotton field.

On DOY 210, the estimated ET error for the fully irrigated corn was 0.5 mm d^-1^ (6.0% error), for irrigate silage corn the error was -8.8%, -11.4% for irrigated cotton, and 0.8 mm d^-1^ or 32.0% for the limited irrigated cotton. In general, ET errors for DOY 210 were lower, -0.2±0.7 mm d^-1^ (-4.7±9.4%) excluding the large error over the limited irrigated cotton field.

**Figure 2.**
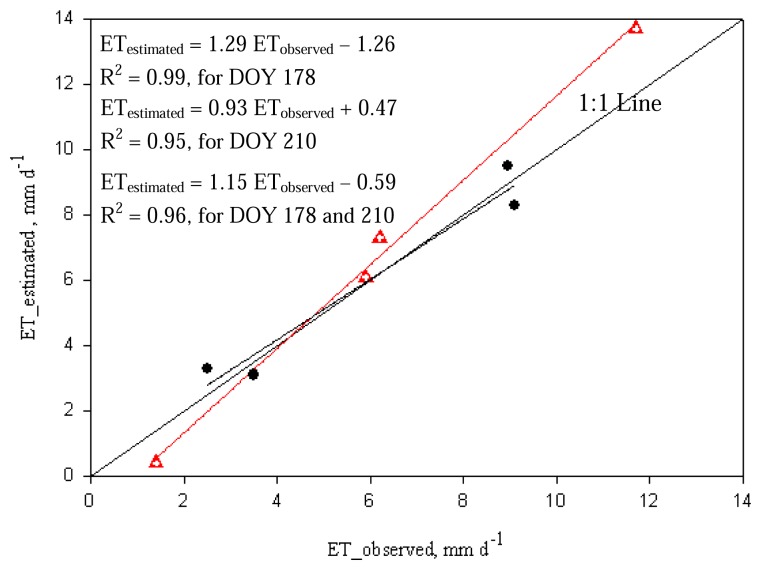
Comparison between METRIC (estimated) and soil water balance (observed) based ET_d_ for two days, June 27 (DOY 178, triangles) and July 29 (DOY 210, circles) of 2005.

Including all data from both days, errors were 0.3±1.0 mm d^-1^ (-1.9±15.5%). However, the RMSE was reduced to 12.4% when the limited irrigated cotton field with low biomass was excluded. Figure 2 shows the graphical comparison and linear regression curves/equations of estimated with measured ET values for both DOYs 178 and 210.

Larger ET estimation errors on the limited irrigated cotton field may be due to late planting in the season, i.e. this field had low biomass with partial canopy cover and high surface temperatures (dT ∼ 4 K). In another study, discrepancies were reported for high surface temperature values, between airborne remotely sensed surface radiometric temperature (MODTRAN [28] calibrated) and those temperatures measured by ground infrared thermometers (IRTs) on corn and soybean fields [29]. It is plausible that the thermal band calibration developed by Tasumi [20] behave similarly for high surface temperatures (>30°C). Thus, larger errors would be expected for low biomass (LAI < 1.5) and/or near bare soil field conditions. Another reason may be related to different surface types (i.e., bare soil vs. irrigated crops) having different relationships between instantaneous LE and daily ET. The METRIC's daily ET scaling mechanism was based on ET_r_F, which may not represent available energy at pixels with low to practically no available crop/water presence, possibly due to different albedo, surface thermal emissivity, surface roughness length, actual relationship between dT and T_s_ being not linear, as well as errors inherent in computing G. In another study, it was found that the evaporative fraction [LE/(R_n_-G)] scaled instantaneous LE to daily ET more accurately for bare soil compared to the ET_r_F approach [30].

Lower ET estimation errors were found on the fully irrigated corn field which had an ET rate closer to the alfalfa reference crop. Some discrepancy on ET may be due to the fact that the cold pixel(s) should be selected in a field with a crop with bio-physical characteristics similar to the alfalfa reference crop, i.e., similar biomass, height, no water stress, disease or lacking nutrients. However, errors could be introduced when the satellite image does not contain such crop conditions.

Moreover, ET_cold_ is assumed by METRIC as 1.05 ET_r_ and it may happen that the selected cold pixel belongs to a crop with a crop coefficient (K_c_ = ET_c_/ET_r_; where ET_c_ is crop ET) that is different than 1.05 at the time of the image acquisition. In our case, the cold pixel was located on an irrigated corn field. Other crops had lower LAI values than most corn fields and had not reached full cover conditions.

In general, the results presented in Table 2 agreed with results obtained in [30], in which the researchers used the standardized ASCE-PM grass reference ET (ET_o_F) to scale daily ET from one-time-of-day 0.5 h ET, and lysimeter data. They observed ET_d_ underestimation errors within 10% for ET>6 mm d^-1^, RMSE of 0.33 to 0.46 mm d^-1^ or errors within 20% for ET values between 3.9 to 5.8 mm d^-1^, and >20% for ET_d_ values ranged 0.4-3.2 mm d^-1^. Their study was conducted on fully irrigated alfalfa, partially irrigated cotton, dryland grain sorghum and bare soil (tilled fallow sorghum).

METRIC captured the difference in water management between the fully irrigated corn field and the somewhat water stressed silage corn. On DOY 178, the predicted ET for fully irrigated grain corn was almost double of that for silage corn (Table 2). This result was supported by other study [14] where it was shown that the amount of water applied to the grain corn as irrigation and rainfall was in excess of the corn potential ET (PET) as calculated by TXHPET. For DOY 210, the actual silage corn ET was similar to that of the fully irrigated corn, i.e. an indication of no crop water stress. Under this condition the ET estimation error was lower than for DOY 178.

**Figure 3.**
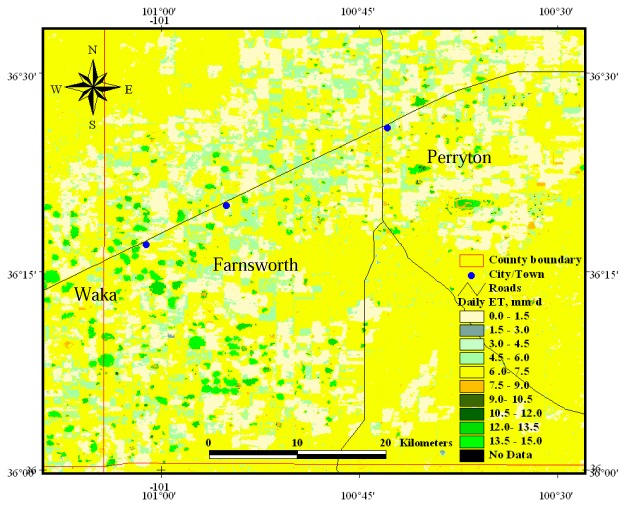
Spatially distributed daily ET for Ochiltree County on DOY 187.

Regional ET_d_ values for Ochiltree County are shown in Figure 3, where bright green fields are high ET rates mainly found on center pivot irrigated corn and soybean fields. Irrigated corn had the highest ET rate, 10.7±3.4 mm d^-1^, i.e. varying from 7.3 to 14.1 mm d^-1^. This result is in excellent agreement with a 3-yr study performed in the same location (Bushland-TX) as described in [31] and [32], where the authors reported that the average measured ET for well irrigated corn, on large monolithic weighing lysimeters, exceeded 10 mm d^-1^ (with a maximum slightly exceeding 14 mm d^-1^) in mid and late June, when monthly average U were 4.0-5.5 m s^-1^. They indicated that crop growth and yields were similar on both the lysimeters and the fields and were representative of normal regional corn production.

Overall, the daily ET results estimated on four commercial agricultural fields indicate that METRIC performs well for the advective conditions of the NTHP. However, larger errors were found for fields with low biomass (<1.5 m^2^ m^-2^). A common standard error for ET prediction equations based on weather data using Penman or Penman-Monteith type equations is as much as 10% of daily estimates [33]. Similarly, using an alfalfa based Penman-Monteith reference ET equation against lysimeter measured alfalfa ET found out that ET estimates using daily weather data underestimated daily ET by about 0.5-0.6 mm d^-1^ on average (5-7% bias) [34].

## 3. Conclusions

METRIC, a remote sensing based ET algorithm was applied to the THP using two Landsat 5 TM images acquired on DOY 178 and 210. Estimated daily ET for well-irrigated, high biomass (LAI > 3.0 m^2^ m^-2^), grain corn resulted with relatively low estimation errors on both days, i.e. 2.0 mm d^-1^ (17.1%) and 0.5 mm d^-1^ (6.0%) respectively. Errors were larger, -71.4 and 32.0% for cotton fields with low biomass (LAI of 0.2 and 1.5 m^2^ m^-2^ respectively) and higher canopy/surface temperatures.

Comparing ET estimates to measurements made on four fields on DOY 178 and 210, the ET_d_ prediction error was 0.3±1.0 mm d^-1^ or -1.9±15.5% (MBE±RMSE). It is possible that the 5% increment over the hourly alfalfa reference ET (ET_r_), suggested in METRIC for the instantaneous ET rate estimation on the cold pixel, might have contributed to the overestimation of ET on well water crops.

For crops/fields displaying low soil-water status, low biomass and high surface temperatures, it is suggested that the following be further researched in order to improve the estimation of small ET rates: atmospheric interference (effects) corrections on the “at-sensor” surface brightness temperature, surface thermal emissivity, relationship between dT and T_s_ (maybe better characterized by a non-linear function), as well as the extrapolation mechanism to obtain daily ET values from instantaneous LE estimates.

In general, METRIC estimated distributed daily ET with relatively low prediction errors for the advective condition encountered in the NTHP. These errors were of similar magnitude of errors reported in the literature for weather station data dependent ET models. Although METRIC is a promising tool for mapping ET accurately in the NTHP, additional evaluation is needed under a variety of crop/weather conditions to fully assess its capability to accurately estimate spatially distributed ET values.

## Figures and Tables

**Table 1.** METRIC input table for H determination.

**Variable**	**Units**	**Cold Pixel_178_**	**Hot Pixel_178_**	**Cold Pixel_210_**	**Hot Pixel_210_**

Elevation	M	907	907	907	907
ET_r_F	-	1.05	0	1.05	0
T_s_	K	291.7	308.0	291.6	315.1
R_n_	W m^-2^	695.0	532.0	692.4	577.0
G	W m^-2^	61.1	106.4	27.8	139.5
Z_om_	m	0.13	0.01	0.125	0.007
U(200 m)	m s^-1^	14.4	14.4	5.9	5.9

**Atmospheric stability corrected values**					

r_ah_	s m^-1^	9.5	10.7	22.8	14.6
u*	m s^-1^	0.78	0.62	0.33	0.35
L__MO_	m	241.2	-44.2	162.4	-7.4
dT	K	-1.36	4.43	-0.36	6.55
LE	W m^-2^	788.4	0.0	680.9	0.0
H	W m^-2^	-154.5	425.6	-16.3	437.5

Note: The subscripts 178 and 210 on the Hot/Cold pixel headings indicate the DOY.

**Table 2.** METRIC and soil water content balance based daily ET (ET_d_).

	**Soil water balance**		**METRIC**		**ET_d_ Difference** **(DOY 178)**		**ET_d_ Difference** **(DOY 210)**	

**Crop**	ET_observed_ (DOY 178)	ET_observed_ (DOY 210)	ET_estimated_ (DOY 178)	ET_estimated_ (DOY 210)	error	error	error	error

	mm d^-1^	mm d^-1^	mm d^-1^	mm d^-1^	mm d^-1^	%	mm d^-1^	%

Fully irrigated corn	11.7	9.0	13.7	9.5	2.0	17.1	0.5	6.0
Irrigated silage corn	6.2	9.1	7.3	8.3	1.1	17.7	-0.8	-8.8
Limited irrigated cotton	1.4	2.5	0.4	3.3	-1.0	-71.4	0.8	32.0
Irrigated cotton	5.9	3.5	6.1	3.1	0.2	3.4	-0.4	-11.4

MBE =					0.6	-8.3	0.0	4.5

RMSE =					1.3	42.6	0.8	19.9

## Appendix A

### Gypsum Block Soil Moisture Readings

Commercially available hand-held gypsum blocks soil moisture meters (tester) do not measure directly in terms of soil water potential but instead they provide a dimensionless relative scale ranging from 0 to 100 units. The Delmhorst gypsum block soil water meter unit KS-D1, used in this research, has its operating instructions manual posted on line at http://www.delmhorst.com/pdf/ks_d1.pdf. The manual provides a curve relating gypsum blocks electrical resistances (ohms) to soil moisture tension (bars) and to meter readings (0-100) in its Figure 1. Lower meter readings translate into dry soil and larger readings into wetter soils. Four (4) meter units read is equivalent to 15 bars of soil water tension while 96 units read equals 0.3 bars. Figure 2, of the same manual, shows the curve relating meter readings to available soil moisture and approximate water used in percent, e.g., 90 meter reading units means 75% of available soil moisture and to approximately 25% of water used. Finally, to obtain the volumetric soil moisture content (VSMC) level or the water depth per soil depth one must multiply the available soil moisture fraction (from the meter) by the soil water holding capacity (volume base). The soil water holding capacity is also called soil available water capacity or crop soil extractable moisture (water content at field capacity minus that at permanent wilting point). In our case, these values were obtained from soil surveys available on-line by geographic location at: http://websoilsurvey.nrcs.usda.gov/app/.

## Appendix B

### Root Zone Soil Water Content Computation

There were three GB-1 Gypsum blocks sensors installed at a depth of 0.3, 0.6, and 0.9 m. These sensors monitored the amount of water per each soil layer. Water depth amounts (mm) per layer (LWD) were computed by multiplying the layer VSMC (in units of mm m^-1^) by the layer thickness, i.e. 0.30 m. Finally, the root zone VSMC or θ_i_ was calculated by adding up the individual layer's LWD values, and the result was reported as water depth equivalent (mm) per 0.9 m of soil depth.
